# Solvent free UV curable waterborne polyurethane acrylate coatings with enhanced hydrophobicity induced by a semi interpenetrating polymer network

**DOI:** 10.1038/s41598-025-04739-1

**Published:** 2025-07-01

**Authors:** Ali Reza Banan

**Affiliations:** Department of Organic Chemistry, Imam Hossein University, Tehran, Iran

**Keywords:** Waterborne polyurethane-acrylate, Semi interpenetrating polymer network, Urethane dimethacrylate, Design of experiments, UV-curing, Polytetrahydrofuran, Green chemistry, Materials chemistry, Polymer chemistry

## Abstract

This study presents the synthesis and characterization of solvent-free, UV-curable waterborne polyurethane-acrylate (WPUA) coatings using a semi-interpenetrating polymer network (sIPN) technique, aiming to develop durable, high-performance eco-friendly coatings. The WPUA formulations consisted of a urethane backbone created from polytetrahydrofuran (PTHF) and isophorone diisocyanate (IPDI), combined with urethane dimethacrylate (UDMA) to introduce acrylate functionality. The synthesized coatings were thoroughly analyzed to assess their morphology, thermal stability, spectroscopic characteristics, water absorption, adhesion strength, and mechanical properties. The design of experiments (DoE) approach was applied to optimize film characteristics, particularly tensile strength. Mechanical testing revealed that incorporating UDMA into the WPUA matrix significantly improved tensile strength, increasing from 4.1 MPa to 8.8 MPa in the optimized formulation. The increase in the cross-cut adhesion test rating from 3B to 5B demonstrates that UV curing significantly enhanced adhesion. Furthermore, the addition of UDMA increased hydrophobicity, reducing water absorption and raising the water contact angle from 67° to 83°.These results indicate that the UV-cured WPUA films possess superior mechanical properties, thermal stability, and water resistance, making them viable options for sustainable coating applications.

**Supplementary Information:**

The online version contains supplementary material available at 10.1038/s41598-025-04739-1.

## Introduction

Growing environmental awareness in recent years has captured the attention of scientists. Waterborne polyurethane (WPU) is extensively utilized in several industries because of its biocompatibility and outstanding characteristics^1,2^. WPU materials have extensive applications in coatings^3,4^adhesives^5^elastomers^6^ antimicrobial behaviors^7,8^and various other industries^9^. Waterborne polyurethane dispersions consist of polyurethane particles that are dispersed in a continuous water phase, forming a binary colloidal system. The primary objective of manufacturing water-based polyurethanes is to develop polymers with an increased quantity of hydrophilic groups to attain water solubility^10,11^. Due to its intrinsic incompatibility with water, it is necessary to alter the polymer backbone of polyurethane to modify and enhance certain properties of water-based polyurethanes, such as tensile strength, surface characteristics, elongation at break, and solvent resistance, a hybrid physical blend consisting of polyurethane and acrylic or vinyl polymers has been proposed^12^. The integration of WPU with UV-curable acrylates presents considerable difficulties owing to the hydrophobic characteristics of acrylates and the polar, water-compatible composition of WPU. This incompatibility frequently results in phase separation, instability in dispersion, and restricted UV-curing efficacy^13^. Furthermore, UV polymerization is acutely sensitive to oxygen and moisture, which can impede radical generation and lead to inadequate curing, particularly at the surface. Hydrophilic groups are crucial for dispersion stability yet harmful to water resistance, making it fundamentally challenging to attain both in a single formulation without targeted design efforts^14,15^.

The semi-interpenetrating polymer network (sIPN) technology efficiently resolves these challenges by demonstrating efficacy in amalgamating polyurethane (PU) with acrylic polymers in composite materials^16^. This technique reduces macroscopic phase separation and improves compatibility between hydrophilic and hydrophobic elements. Research indicates that sIPN systems provide enhanced stability, mechanical strength, and water resistance relative to basic blends^15,17^. Acrylates play a key role in sIPN systems, which have become a prominent area of research in polymer composites. The unique properties of hybrid polymer systems are influenced by the presence or absence of anchoring sites within the polymer structure^18^. These anchoring points can be introduced through grafting or end-capping with acrylates or they may be absent in physical blends and interpenetrating networks^12,19^.

Hydroxyethyl acrylate (HEA) and hydroxyethyl methacrylate (HEMA) are often utilized monomers in the manufacture of end-capped and hybrid polymer networks^20,21^. The WPUA unique segmented structure and acrylate modification enable it to exhibit a wide range of enhanced attributes and performance. It can be efficiently utilized in coating applications^22^.

UV-curing coatings exhibit enhanced toughness, resistance to abrasion and chemical durability in comparison to conventional coatings, owing to their cross-linked structure and robust hydrogen bonding^23^. The introduction of UV-curing technology enhances the physical and chemical qualities of waterborne polyurethane. Acrylate is a common UV-curable matrix resin, and acrylate coatings offer several benefits with their most notable feature being high reactivity^24–26^. Typically, adding acrylate monomers can enhance the resin’s performance, making it suitable for particular applications^27^.

The introduction of hydrophobic components is commonly used to obtain more water-resistant materials^28^. The incorporation of urethane dimethacrylate (UDMA) prepolymer as a hydrophobic and curing agent in water-based polyurethane coatings, which are cured using UV light, leads to several improvements^29^. These include enhanced hydrophobicity, increased mechanical strength, improved chemical and abrasion resistance, and higher UV curing efficiency^30,31^. Additionally, the use of UDMA prepolymer improves adhesion and achieves a desirable balance between flexibility and hardness. As a result, this coating becomes highly durable and protective, making it suitable for a wide range of substrates and harsh environments^32^.

Yunjiao Deng et al.^33^ utilized multiple approaches, including an interpenetrating network (IPN) approach, to synthesize polyurethane-acrylate composites, emphasizing the assessment of their structural and mechanical properties. A novel method is presented for developing a two-component UV-curable aqueous polyurethane-acrylate system based on a semi-interpenetrating polymer network (sIPN) incorporating urethane dimethacrylate (UDMA). This method highlights a solvent-free synthesis and sophisticated experimental design. These systems consist of two components, the first includes a UDMA prepolymer, while the second, composed of isophorone diisocyanate (IPDI), polytetrahydrofuran (PTHF), 1,4-butanediol (BDO), and 2-dimethylolpropionic acid (DMPA), forms the water-based polyurethane. The initial step involves the synthesis of the urethane dimethacrylate prepolymer and the optimization of the reaction conditions using design of the experiment (DoE). The prepolymer was synthesized through a solvent-free reaction at an optimal temperature, and the resulting resin was analyzed using FT-IR^1^, H-NMR, and viscosity measurements. A waterborne polyurethane was synthesized using IPDI, PTHF, BDO, and DMPA. After neutralizing the acidic groups with triethylamine (TEA), the polymer was uniformly dispersed in water, forming a stable waterborne resin dispersion. Subsequently, the two components were combined, resulting in the formation of UV-curing waterborne polyurethane-acrylate by the semi-interpenetrating polymer network (sIPN) method. To evaluate the properties, thermal resistance, mechanical characteristics, water contact angle, water absorption, and FT-IR analysis were conducted.

## **Experimental**

### Materials

Polytetrahydrofuran (PTHF, Mn = 1000 g/mol) was sourced from Sigma-Aldrich. Isophorone diisocyanate (IPDI, 99%) was obtained from Rongrong Chemical Ltd. 2-Hydroxyethyl methacrylate (HEMA, 99%) was supplied by Ruipu New Material Co., Ltd. The photoinitiator, 2-Hydroxy-2-methyl-phenyl-propane-1-one (Darocur 1173, 99%), was provided by Mingda Macromolecule Science and Technology Co., Ltd. Dimethylolpropionic acid (DMPA, 98%), 1,4-Butanediol (BDO, 99%), triethylamine (TEA, 99%), and dibutyltin dilaurate (DBTDL, 98%) were all purchased from Merck.

### Synthesis of urethane dimethacrylate (UDMA) resin

**Fig. 1:**
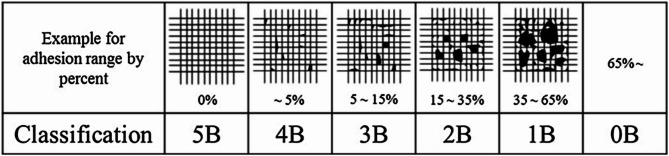
Cross-cut adhesion scale for coatings according to ASTM D3359 standard.

UDMA was synthesized by reacting IPDI with HEMA in the presence of DBTDL as a catalyst. To commence the solvent-free reaction, 7.1 g (32 mmol) of IPDI and 0.03 g of DBTDL were placed in a 25-mL three-necked flask with a magnetic stirrer, nitrogen inlet, and dropping funnel. The flask was submerged in an ice bath, and then HEMA (3.7 g, 32 mmol) was slowly added to the mixture at 0 °C while being stirred continuously for 4 h. The temperature was then elevated to 80 °C, and the mixture was agitated for an extra 12 h until the desired isocyanate value was verified using titration, resulting in the formation of the IPDI-HEMA (UDMA) adduct. The synthesis steps are illustrated in Fig. 1.

### Optimization of reaction parameters by DoE approach

The effect of various factors and the most dominant parameters affecting waterborne polyurethane-acrylate quality was determined using the DoE approach^34,35^. The Taguchi methodology uses the factorial design to organize the experiments in an orthogonal arrays-based approach. The amount of stress in WPUA has been affected by four factors, such as DMPA, UDMA, IPDI, and PTHF amounts. The values for each factor were carefully chosen as the experiment was based on the findings of previous literature^36,37^. Three levels were given to each factor so that a clear and precise distribution of values could be seen, as illustrated in Table 1.

**Table 1:** Different levels of each factor and their corresponding values (screening process parameters).

Factor (gr)	Symbol	Level 1	Level 2	Level 3
Dimethylolpropionic acid	(DMPA)	0.5	1	1.5
Urethane dimethacrylate	(UDMA)	0.5	1	1.5
Isophorone diisocyanate	(IPDI)	4	6	8
Polytetrahydrofuran	(PTHF)	7	14	28

Nine experiments were performed with a conventional orthogonal array (OA_9_) built according to the Taguchi method. The data underwent statistical analysis using Minitab. The process yields dependable and precise outcomes. Three levels were employed to analyze the impact of each examined condition with the designated variables. By conducting the specified tests and analyzing the results, the optimal formulation referred to as number 10 was identified, which includes the best levels for each factor in WPUA production (Table 2).

### Synthesis of waterborne polyurethane-acrylate

Figure 1 illustrates the solvent-free synthesis process of WPUA, wherein PTHF 14 g (16.1 mmol) and IPDI 6 g (27 mmol) were introduced into a 100 mL three-necked reactor, followed by the addition of DBTDL (20 µL) and subsequent heating to 70 °C. The mixture was agitated with a mechanical stirrer in a nitrogen environment for four hours. DMPA 1 g (7.5 mmol) and BDO 0.3 mL (3.4 mmol) were incorporated into the mixture and agitated at 80 °C for 2 h, with the reaction continuing until the NCO concentration achieved the theoretical value, indicating the completion of the reaction. The resultant NCO-terminated polyurethane prepolymer was cooled to 40 °C. Subsequently, UDMA 1.5 g (3.1 mmol) was incorporated and permitted to blend adequately with WPU, resulting in a physical mixing of WPU and UDMA at a steady temperature for 3 h to synthesize the waterborne polyurethane-acrylate. A mixture of distilled water and TEA was gradually poured into the dropping funnel under a mechanical stirrer at 40 °C to neutralize the carboxylic acid groups of DMPA for one hour. Finally, a milky solution containing WPUA was acquired.

**Scheme 1:**
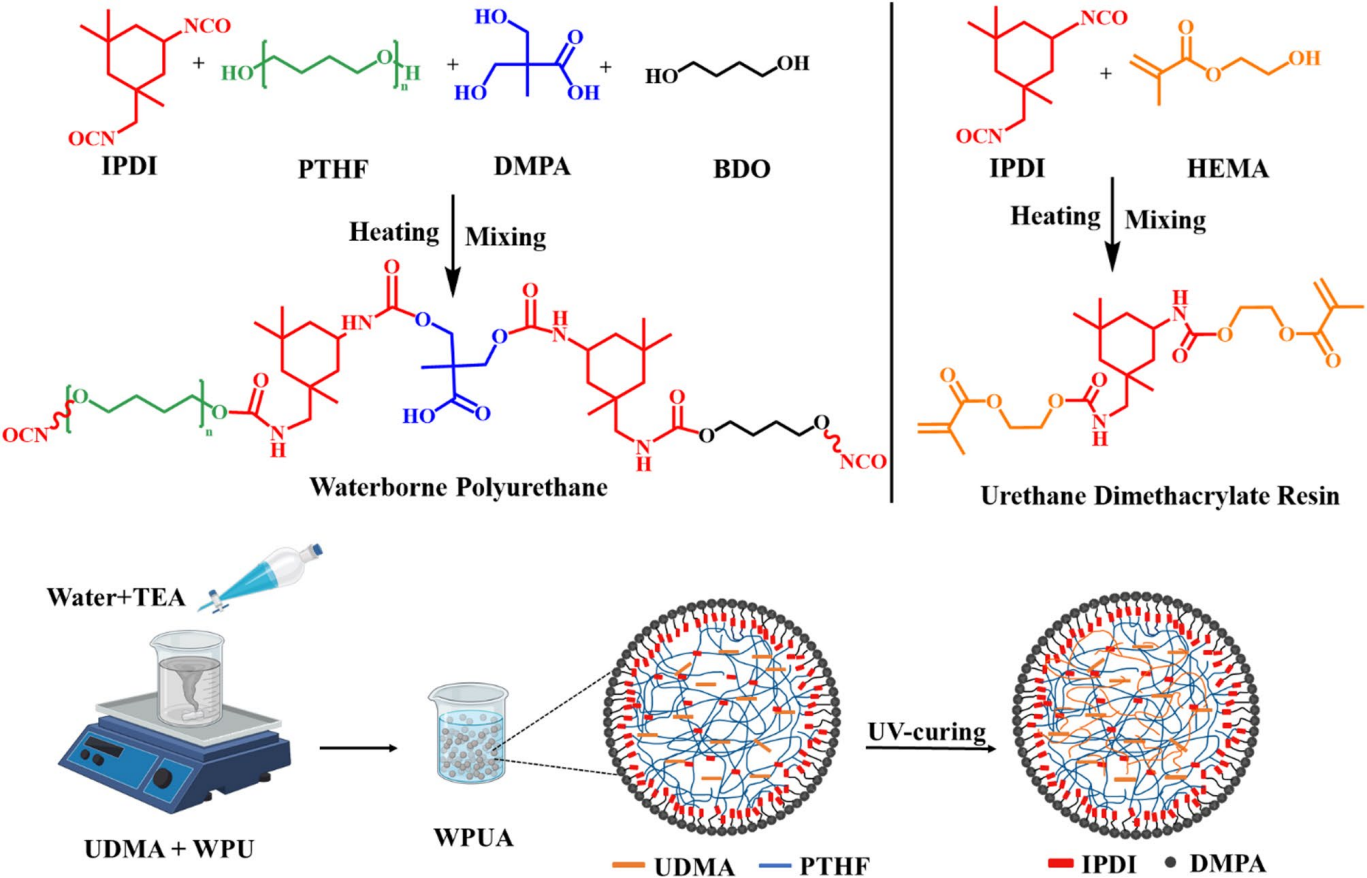
Synthesis steps UV-curable waterborne polyurethane.

### Preparation of UV-cured WPUA film

The photoinitiator (Darocur 1173) was added to the WPUA with stirring at 100 rpm for 10 min. The WPUA film was produced by pouring it into Teflon molds measuring 10 cm × 10 cm × 0.3 mm, and subsequently stored in a dark box at ambient temperature for 24 h. The dried WPUA film samples were then cured under a 400 W-powered UV source, the distance from the center of the UV lamp to the WPUA film samples was set at 10 cm.

### Instrumentations and measurements

The emulsion’s particle size and distribution were analyzed via dynamic light scattering (DLS) utilizing the Brookhaven 90 Plus Laser Particle Analyzer. The samples underwent dilution with deionized water at ambient temperature, and each sample was measured three times to ensure precision. Fourier-transform infrared spectroscopy (FT-IR) spectra were acquired in ATR mode and the spectra were obtained within a wavenumber range of 650 to 4000 cm^− 1^, with a resolution of 8 cm^− 1^, employing a thin polymer film for the analysis. The film’s contact angle was assessed utilizing a Contact Angle Goniometer (DSA30, Kruss Co). The determination of surface energy was conducted through contact angle experiments employing two liquids: water and ethylene glycol. The surface energy was determined using the Owen-Wendt theory, applying Eqs. 1, 2. For each sample, three independent measurements were performed to verify the reliability and accuracy of the results.

1 $$\:\frac{{\upsigma}_{\text{L}}{(\cos \theta +1)}}{\sqrt[\text{2}]{{\upsigma}_{\text{L}}^{\text{D}}}}{=\:}\sqrt{{\upsigma}_{\text{S}}^{\text{P}}}{.}\frac{\sqrt{{\upsigma}_{\text{L}}^{\text{P}}}}{{\upsigma}_{\text{L}}^{\text{D}}}{+\:}\sqrt{{\upsigma}_{\text{S}}^{\text{D}}}$$.

This equation has the linear form y = mx + b, wherein:

2 $$\:\text{y}\:=\:\frac{{\sigma\:}_{L}(cos\theta\:+1)}{\sqrt[2]{{\sigma\:}_{L}^{D}}}.m=\sqrt{{\sigma\:}_{S}^{P}},x=\:\frac{\sqrt{{\sigma\:}_{L}^{P}}}{\sqrt{{\sigma\:}_{L}^{D}}},\:b=\:\sqrt{{\sigma\:}_{S}^{D}}$$.

Here θ represents the contact angle, σ_L_ = represents the overall surface tension of the wetting liquid, $\:{{\upsigma\:}}_{\text{L}}^{\text{D}\:}$= denotes the dispersive component of the surface tension of the wetting liquid, and $\:{{\upsigma\:}}_{\text{L}}^{\text{P}\:}$= signifies the polar component of the surface tension of the wetting liquid, $\:{{\upsigma\:}}_{\text{S}}^{\text{D}\:}$= dispersive component of the surface energy of the solid, and $\:{{\upsigma\:}}_{\text{S}}^{\text{P}\:}$= represents the polar component of the surface energy of the solid^38^. During contact angle measurements, these components help quantify the interactions between the liquid and the solid surface.

Tensile testing of the films was performed with a universal testing apparatus (Instron 3365 tensile tester) at a cross-head speed of 20 mm/min and a temperature of 25 °C, adhering to ASTM D638 criteria. Each sample underwent triplicate testing, and the optimal value from these tests was documented for analysis. Thermogravimetric analysis (TGA/DTG) was conducted with a Pyris thermogravimetric analyzer. Film samples weighing 3–4 mg was positioned in a platinum pan and subjected to heating in a nitrogen environment at a rate of 10 °C/min, ranging from 25 °C to 600 °C. The morphology of the WPUA films’ surfaces was analyzed using scanning electron microscopy (SEM) to examine the changes in the film structure before and after UV curing. This investigation facilitated a comprehensive evaluation of the surface properties and structural alterations that transpire during the curing process.

The water absorption of the WPU and WPUA films, both before and after the curing process, was evaluated by weighing a dried film sample and immersing it in a container of water for one week. Each day, a sample was removed from the water, and the surface moisture was blotted dry before weighing. The water absorption (W) was calculated using the Eq. 3:

3 $$\:\text{W}\left({\%}\right){=\:}\frac{{\text{W}}_{\text{t}} - {\text{W}}_{\text{0}}}{{\text{W}}_{\text{0}}} \times {100}$$.

W_0_ and W_t_ denote the mass of the film before immersion in water and the mass of the film at time t, respectively.

The total solids content (TSC) of the WPUA mixture was assessed by the ISO 124:1997 standard procedure. During this procedure, one gram of the material was measured before and after drying in an oven at 70 °C. The weighing procedure was reiterated until a stable weight was achieved. The TSC was subsequently determined by calculating the weight differential before and after the drying process. To evaluate the stability of dispersions, samples were positioned undisturbed in sealed clear containers. The physical appearance of materials was visually assessed under ambient settings for a duration of 1 to 3 weeks.

For the adhesion assessments of WPU and UV-curable WPUA films applied onto stainless steel substrates, both the pull-off strength and cross-cut adhesion were evaluated following ASTM standards D4541 and D3359, respectively (Fig. 1). The cross-cut test, which assesses the proportion of 1 × 1 mm squares of the polymeric coating removed from the substrate by adhesive tape, was conducted using a multi-blade cutting device (model 0302001 from Neurtek Instruments S.A., Eibar, Spain). The device made six parallel cuts on the coating surface, with a second set of perpendicular cuts applied to create a grid pattern. Tesa adhesive tape was then applied to the grid, and the tape was pulled off, allowing for the counting of the removed coating squares. Three replicates of this test were performed, and the results were averaged, with adhesion values ranked according to the ASTM D3359 scale.

For the pull-off strength analysis, the Positest-AT-Automatic (Defelsko Co., NY) was used on the WPU and UV-curable WPUA films. The mold used had an area of 12.7 mm^2^, and surface pollutants were eliminated using a triadic cleaning protocol in a detergent solution. A stud was attached to the polyurethane film’s surface using a thermally curable epoxy adhesive, and pull-off strength values were recorded. This method provided a comprehensive assessment of the adhesion properties of the coatings on stainless steel substrates.

## Results and discussion

### Design of experiments for synthesized WPUA film

Experimental design is a critical subject currently addressed across multiple sectors and in laboratory experiments. Employing DoE approaches facilitates the identification of the interacting impacts of various elements that affect the output results of measurements. The Taguchi design of experiments was used to determine the factors influencing the stress of WPUA films. The data obtained from this approach were analyzed to identify the most significant factors and establish the optimal levels of each factor relative to the objective function (Table 2).

**Table 2:** Optimization of synthesis procedure using the OA9 matrix.

Test number		Factors							Results	
		DMPA(g)		UDMA (g)		IPDI (g)	PTHF (g)		Stress (MPa)	
1		0.5		0.5		4	7		4.1	
2		0.5		1.0		6	14		8.3	
3		0.5		1.5		8	28		6.7	
4		1.0		0.5		6	28		5.6	
5		1.0		1.0		8	7		7.4	
6		1.0		1.5		4	14		6.0	
7		1.5		0.5		8	14		6.2	
8		1.5		1.0		4	28		4.0	
9		1.5		1.5		6	7		7.2	
10*		1.0		1.5		6	14		8.8	

* Indicates an optimized formulation, all component values in grams; stress values represent tensile strength in MPa.

**Fig. 2:**
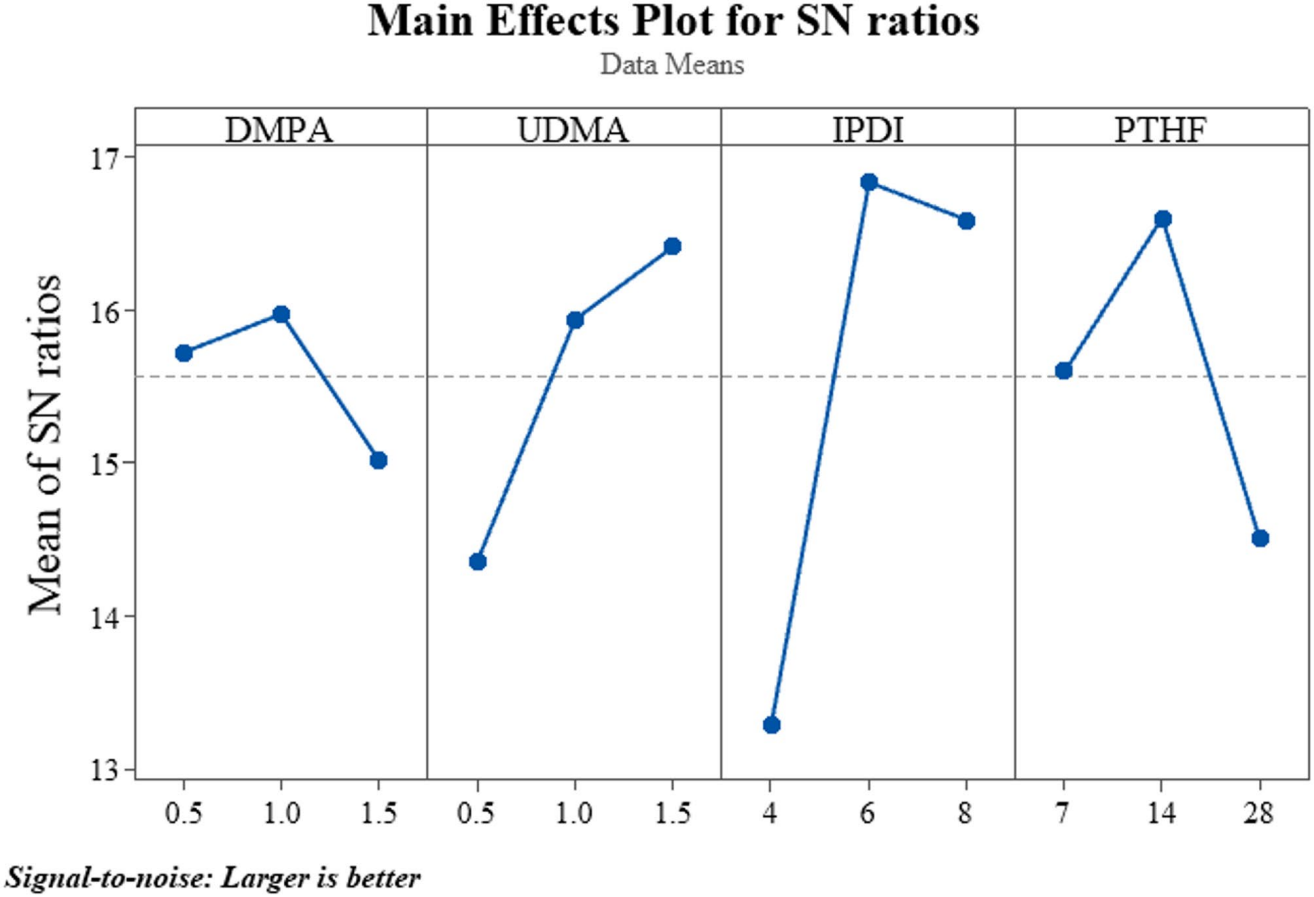
The optimal values of factors by signal-to-noise ratio curves. Minitab Statistical Software version 22 was used to generate the plot (https://www.minitab.com/).

The Taguchi analysis indicated that the most significant factor influencing the experiment’s outcome was the quantity of IPDI, with level 2 (6 g) identified as optimal. The second critical factor was the quantity of PTHF, with the ideal level being 2 at 14 g. UDMA at 1.5 g (level 3) and DMPA at 1 g (level 2) ranked third and fourth, respectively, in terms of their impact on the polymer film’s tensile strength. To further validate the experimental results, an additional sample, designated as Sample 10, was synthesized using the optimal levels of the identified factors. This sample will serve as the primary reference in subsequent reaction processes (Fig. 2).

### Response surface methodology (RSM)

**Fig. 3:**
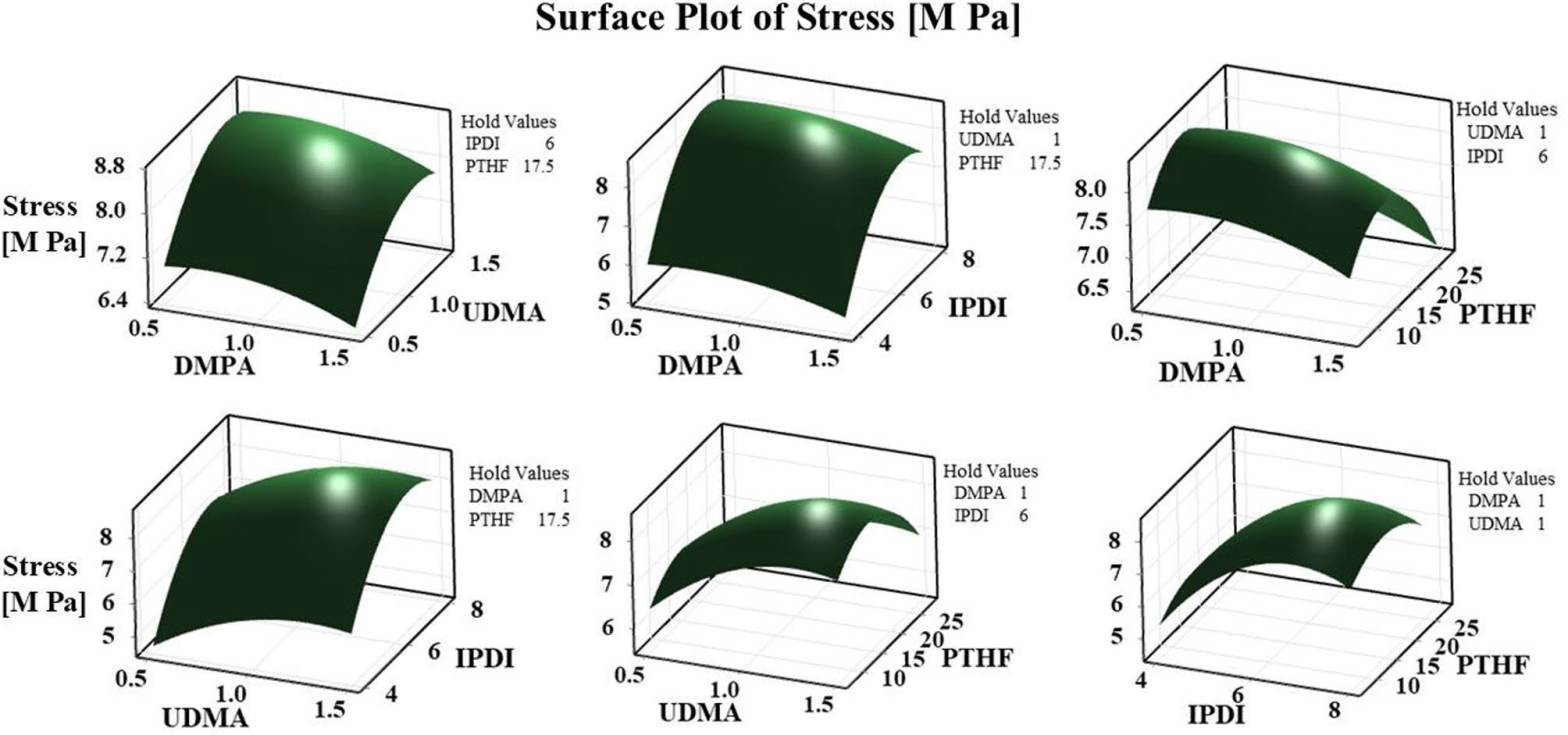
A response surface model (3D surface plot) to assess the impact of factors on the tensile strength of the WPUA film. The plot was generated using Minitab Statistical Software version 22 (https://www.minitab.com/).

To investigate the relationship between the selected parameters and the strength of the waterborne polyurethane (WPU) film, Response Surface Methodology (RSM) was applied^39^. This statistical approach defines IPDI, PTHF, UDMA, and DMPA as independent variables, with the tensile strength of the WPU film serving as the dependent variable. The 3D plots clearly illustrate the impact of each parameter on the result. Each figure illustrates the influence of two variables on the stress of the synthesized film, while maintaining the other two variables constant, facilitating a comprehensive examination of both individual and interacting effects. As shown in Fig. 3, the film’s tensile strength reaches its maximum value as the factors approach their optimal levels. These visual representations provide a clear illustration of the correlation between the variables and the stress of the WPUA films, demonstrating how fluctuations in IPDI, PTHF, UDMA, and DMPA influence the mechanical performance of the films.

**Fig. 4:**
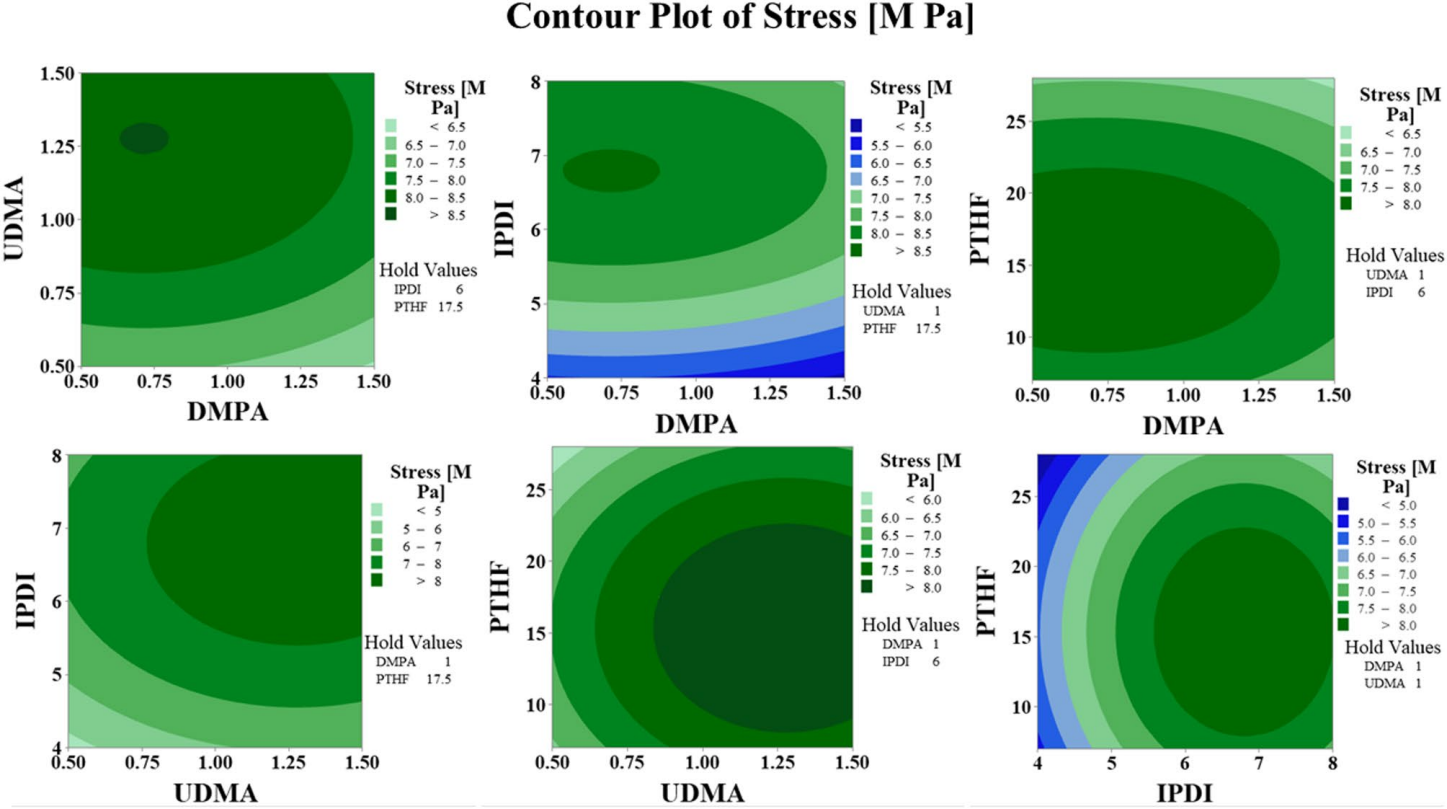
Contour plot based on the response surface methodology (RSM) model to assess the impact of factors on the tensile strength of the WPUA film. The plot was produced using Minitab Statistical Software version 22 (https://www.minitab.com/).

Figure 4 presents 2D contour plots generated from the RSM prediction models. These plots illustrate the effects of different parameters on the final film stress. The color gradient, extending from minimum to maximum, distinctly illustrates the impact of various factor combinations on the mechanical properties of the films^40^. An increase in UDMA significantly enhances the stress resistance of WPUA films. This is attributable to the UDMA function in augmenting the film’s cross-link density, hence yielding superior mechanical properties. Furthermore, optimizing the average concentration of PTHF enhances mechanical characteristics by achieving an equilibrium between flexibility and strength within the polymer matrix. The interactions between UDMA and PTHF are well depicted in the contour plots, providing insights into how adjusting these parameters can improve film performance regarding stress resistance and durability.

### WPU and WPUA properties

WPU and WPUA based on UDMA were successfully synthesized via step-growth polymerization. The properties of both WPU and the new series of WPUA are summarized in Table 1. At 25% total solid content (TSC), WPU exhibited a milky appearance, while WPUA displayed a milky yellow appearance at 31% TSC.

The average particle size of WPU coatings is determined by their intended application. Larger particles tend to dry more quickly, whereas smaller particles are better suited for penetrating surface pores. Several factors, including the NCO ratio^41^the amount of chain extender^42^the molecular weight and nature of the polyol^43^and the degree of neutralization^44^ influence the particle size of WPUA. In this research, the particle sizes of WPU and WPUA were compared and analyzed. The particle size of WPU dispersions increased from 82 nm to 120 nm with the introduction of UDMA, as seen in Table 3. This increase is attributed to the hydrophobic nature of UDMA prepolymer. When the acylated prepolymer is physically mixed with WPU, UDMA tends to localize in the core, while WPU forms the shell of the nanoparticles upon the addition of water to the polymer mixture, resulting in the formation of larger polyurethane particles.

The stability of aqueous polyurethane dispersions is a crucial element in their production and processing. Dispersion systems with reduced particle sizes (< 200 nm) are typically regarded as storage-stable and are favored for their elevated surface energy, which facilitates effective film formation. Table 3 demonstrates that both WPU and WPUA dispersions exhibited stability for more than two weeks, signifying an acceptable shelf life. The physical appearance of WPU was translucent, whereas WPUA had a milky white hue, both being steady during the observation period. The WPU dispersion exhibited remarkable stability throughout a three-week period, with particle size escalating from 82 nm in the first week to 98 nm in the third week, while preserving an appropriate polydispersity index (PDI). The WPUA dispersion exhibited commendable stability for two weeks, with particle sizes of 120 nm with a PDI of 0.2 in the first week, and 132 nm with a PDI of 0.22 in the second week. By the third week, the WPUA dispersion exhibited symptoms of aggregation and settling attributable to the hydrophobic groups of UDMA, adversely affecting its long-term stability.

**Table 3:** The properties of the WPU and WPUA dispersions.

Sample	TSC		Stability					Average Particle size (nm)					
	%		1 weeks		2 weeks	3 weeks		1 weeks		2 weeks		3 weeks	
								DLS	PDI	DLS	PDI	DLS	PDI
WPU	25		Stable		Stable	Stable		82	0.1	93	0.11	98	0.14
WPUA	31		Stable		Stable	Non-stable		120	0.2	132	0.22	----	----

### The properties of WPUA films

After casting WPUA latexes in a Teflon mold and drying them to a constant weight, the resulting films appeared opaque and non-transparent. The WPUA film exhibited a light-yellow color.

**Fig. 5:**
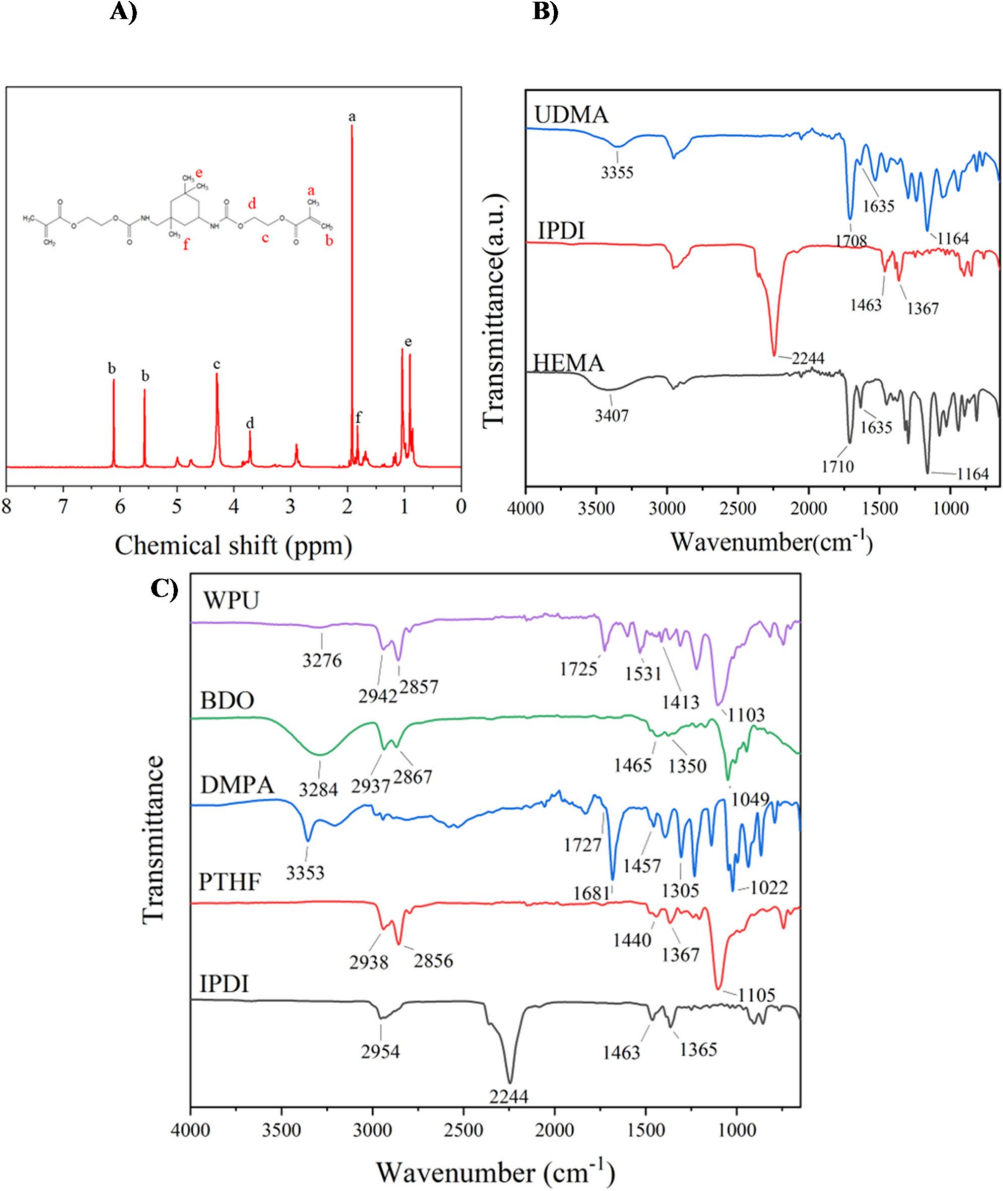
Characterization of UDMA and WPU, (**A**) the^1^H-NMR spectrum of UDMA. (**B**) The FT-IR spectra of UDMA. (**C**) The FT-IR spectra of WPU film.

The^1^H-NMR spectrum of UDMA is illustrated in Fig. 5A. The peaks observed between 0.96 and 2.9 ppm correspond to the methyl (CH_3_) and methylene (CH_2_) protons of UDMA. The proton from the NHCO group appears at 3.7 ppm. The –CH₃ group of UDMA is detected at 0.14 ppm. The peaks at 4.0 and 4.3 ppm are attributed to the methylene protons of HEMA. The characteristic acrylate protons and methyl (CH₃) groups of HEMA are observed at 5.5–6.1 ppm and 1.93 ppm, respectively^45,46^.

Isophorone diisocyanate (IPDI) is an organic chemical predominantly utilized as a precursor in the synthesis of polyurethane. The FT-IR spectrum of IPDI provides essential insights into its functional groupings. The absorption bands at 1365 cm⁻¹ and 1465 cm⁻¹ correspond to the bending vibrations of the methylene (-CH_2_-) and methyl (-CH_2_) groups in the cycloaliphatic ring structure. A distinct band at 2244 cm^− 1^ signifies the N = C = O stretching vibration of the isocyanate (-NCO) group, showing the existence of isocyanate functional groups in IPDI. The band observed at 2954 cm^− 1^ corresponds to C-H stretching vibrations in aliphatic molecules^47^. The FT-IR spectrum of HEMA displays a broad band at 3410 cm^− 1^, indicative of O-H stretching. A strong band at 1710 cm^− 1^ corresponds to C = O stretching in the methacrylate ester, while another at 1635 cm^− 1^ is linked to C = C stretching in the methacrylate group. These bands confirm the existence of hydroxyl and ester groups, along with a double bond in the methacrylate unit of HEMA^48^. The FT-IR spectrum of UDMA shows an absorption band at 3355 cm^− 1^ indicative of N-H stretching in the urethane group, a band at 1708 cm^− 1^ representing C = O stretching in the urethane linkage, and a band at 1635 cm^− 1^ associated with C = C stretching in the methacrylate group (Fig. 5B). The complete disappearance of the band around 2244 cm^− 1^ indicates that the reaction has reached completion and that the -NCO groups have been fully consumed. The peaks validate the effective synthesis of UDMA from IPDI and HEMA.

The FT-IR spectra of PTHF exhibit a band at 1105 cm^− 1^ corresponding to C-O-C stretching, signifying the presence of ether bonds. The bands at 1347 cm^− 1^ and 1440 cm^− 1^ indicate C-H bending in -CH₂- groups, whilst 2856 cm^− 1^ and 2938 cm^− 1^ signify C-H stretching in these aliphatic groups, so verifying the existence of methylene groups and ether linkages^49^. The FT-IR spectra of DMPA have a large band at 3353 cm^− 1^, signifying O-H stretching and the presence of hydroxyl groups. A band at 1681 cm^− 1^ indicates C=O stretching associated with carboxylic acids. The IR spectra of BDO have a large band at 3284 cm^− 1^, signifying O-H stretching and affirming the presence of hydroxyl groups. A band at 1049 cm^− 1^ is indicative of C-O stretching. The absorption bands at 1350 cm^− 1^ and 1465 cm^− 1^ indicate C-H bending. These observations confirm the existence of methylene and hydroxyl groups in BDO. The FT-IR spectra of WPU have a band at 1103 cm^− 1^, which verifies the existence of ether connections (C-O-C stretching). The band at 1413 cm^− 1^ signifies C-H bending in aliphatic groups, while the one at 1531 cm^− 1^ peak denotes N-H bending in urethane linkages. A strong absorption band at 1725 cm^− 1^ is ascribed to C=O stretching in urethane bonds. The bands at 2857 cm^− 1^ and 2942 cm^− 1^ correspond to C-H stretching, while the peak at 3276 cm^− 1^ indicates N-H and O-H stretching, presumably from residual hydroxyl groups^50^ (Figure 5 C).

### The properties of UV-cured WPUA film

The photoinitiator (Darocur 1173) absorbs energy and generates free radicals, which accelerate the polymerization reactions, leading to a rapid increase in conversion. This process initiates the crosslinking reaction during free-radical polymerization. After 4 min of UV exposure, the curing process was completed, resulting in the formation of a sIPN structure in WPUA. The WPUA film was successfully cured, forming networks through photo-crosslinking.

**Fig. 6:**
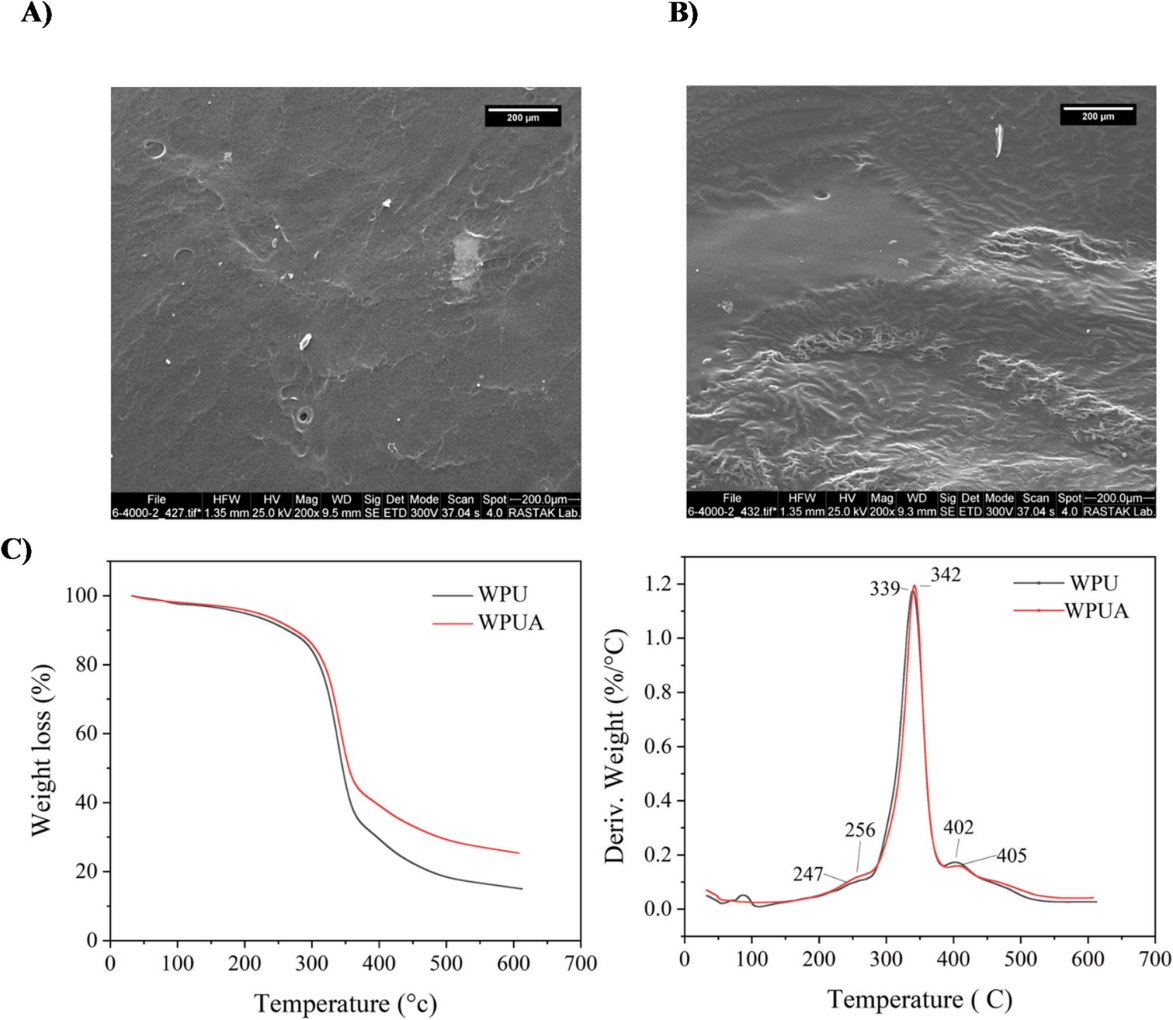
(A) The surface morphology of the WPU film through SEM images, (B) The surface morphology of the WPUA film, providing a detailed view of the film’s structure. (C) The TGA/DTG graph, illustrating the thermal properties of the WPU and WPUA film.

The SEM images of the WPUA film, as shown in Fig. 6 (A, B), reveal that the surface of the WPUA film before UV curing is smooth and uniform. However, after the curing process, the surface became noticeably rougher, although no phase separation was observed. The augmented surface roughness post-UV curing improves adhesion by generating additional mechanical interlocking sites, resulting from hydrogen bonding and the production of extra hard segments during cross-linking.

The thermal degradation behavior of WPU and UV-curable WPUA films based on TGA/ DTG are shown in Figure 6 (C). The thermal degradation of these coatings begins at temperatures above 200 °C, with the WPUA film exhibiting superior thermal resistance compared to the WPU film. This improvement is attributed to the formation of a well-integrated cross-linked network within the final film structure^51^. This cross-linking enhances both the mechanical strength and thermal stability of the polyurethane film, providing better overall performance at elevated temperatures. The DTG curves reveal three distinct decomposition steps. The first decomposition, occurring at approximately 247 °C for WPU, is ascribed to the breakdown of hard segments at the urethane linkages and the release of carbon dioxide from urethane groups. The subsequent breakdown phase, at 339 °C, corresponds to the degradation of soft segment in the WPU film. The final decomposition step occurs around 402 °C, which is likely due to the breakdown of urethane linkages in the polymer matrix. In the case of UV-curable WPUA films, this step is somewhat elevated to a higher temperature due to the presence of UDMA in the film. The shift at 342 °C is likely due to the degradation of hydrogen bonds in the soft segment of WPUA, which contributes to the enhanced thermal stability of the material. The final stage of decomposition, occurring around 405 °C, is attributed to the breakdown of other tightly bonded fragments within the polymer, particularly those connected to the acrylate structure.

**Fig. 7:**
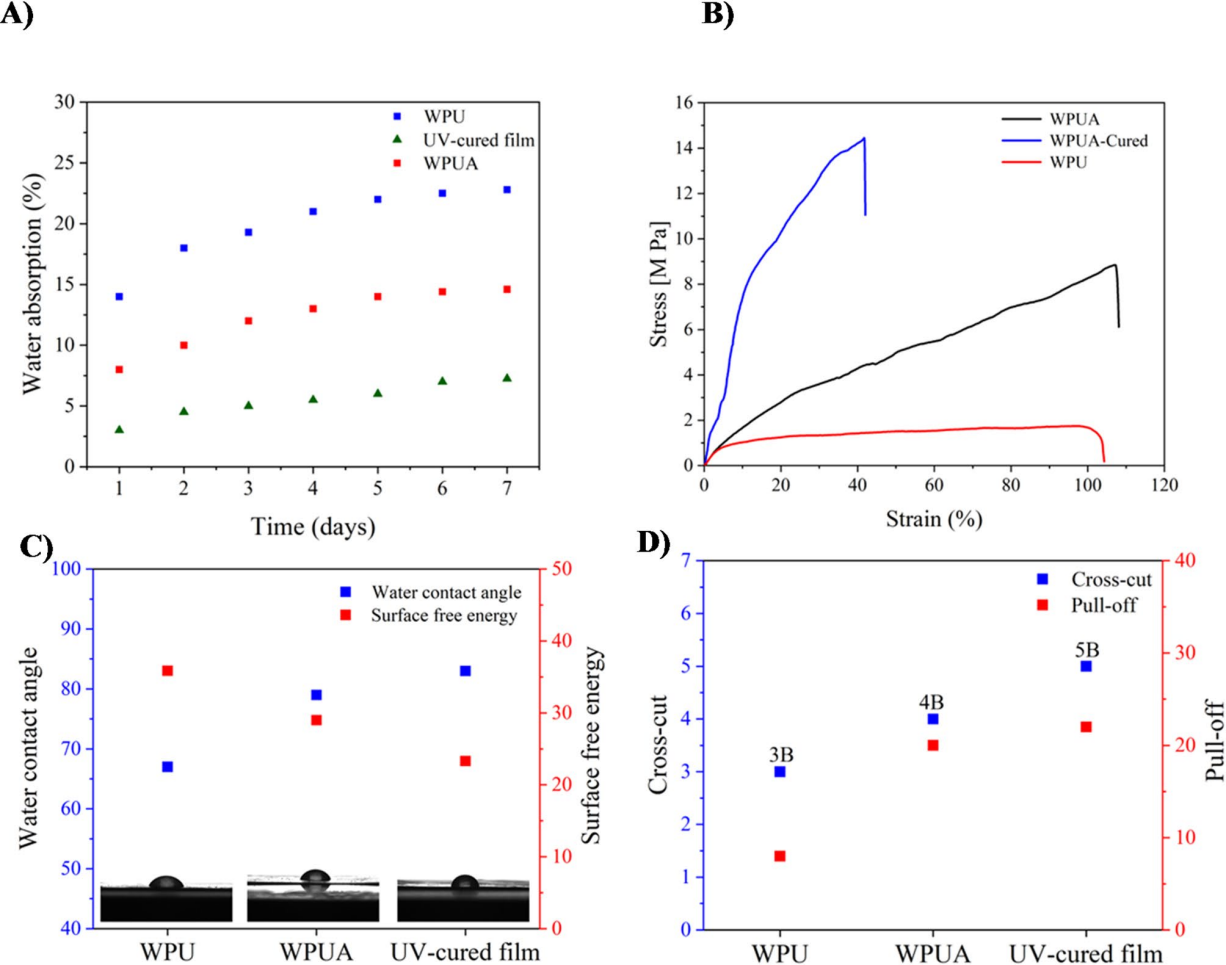
(**A**) Water absorption of the WPU and WPUA films. (**B**) Mechanical performance, was assessed through the stress-strain behavior of WPU and WPUA films. (**C**) Water contact angle and surface free energy of WPU, WPUA, and UV-cured WPUA films. (**D**) Cross-cut adhesion and pull-off adhesion tests to assess the adhesion strength of the films.

The water absorption of WPU and WPUA films was examined, as illustrated in Fig. 7 (A). The WPU film demonstrated greater water absorption than the WPUA film, attributable to the hydrophobic characteristics of UDMA in the structure of WPUA. Integrating this hydrophobic substance into the polyurethane film diminishes its water absorption characteristics. Post-UV curing, the WPUA film exhibited an augmented cross-linking density and a reduced free volume inside the molecular chains. This outcome indicates that the UV-cured WPUA film, with its highly cross-linked network, strengthens the intermolecular interactions, making the molecular chains more compact. As a result, water penetration into the film is significantly reduced, leading to lower moisture absorption in humid environments^52^.

As shown in Table 4, the water absorption of the UV-cured WPUA film markedly diminished in comparison to both the WPU and WPUA films. Following UV curing, the water absorption of the WPU film diminished by roughly 68.2% (from 22.8 to 7.25%), while the WPUA film reduced by around 50.3% (from 14.6 to 7.25%). This reduction underscores the efficacy of UV curing in improving the material’s water resistance.

**Table 4:** Water absorption data for WPU, WPUA, and UV-cured WPUA films at different time points.

Time (days)	WPU (%)	WPUA (%)	UV-cured WPUA (%)
1	14	8	3
2	18	10	4.5
3	19.3	12	5
4	21	13	5.5
5	22	14	6
6	22.5	14.4	7
7	22.8	14.6	7.25

The mechanical performance of the WPU and WPUA films was evaluated by analyzing their stress-strain behavior, both pre-and post-curing film. The impact of UDMA acrylate groups on the mechanical properties of polyurethane films was evaluated to determine the strength and durability of the WPU coatings^53^. This assessment elucidates the impact of UDMA on the mechanical durability of the films. According to the stress-strain curves in Fig. 7 (B), the incorporation of UDMA prepolymer significantly increased the tensile strength, indicating strong compatibility between polyurethane and UDMA. The formation of sIPN structures promotes greater chain entanglement between the polyurethane and UDMA chains. This improved interaction enhances the tensile strength of the final film. Post UV-curing, the WPUA film exhibited enhanced strength attributable to the cross-linking established within the polyurethane network. The tensile strength of the UV-cured WPUA film rose relative to the uncured film, however the elongation at break diminished. The decrease in elongation is characteristic of cross-linked networks, where chain mobility constrained restricts flexibility while improving mechanical strength and durability.

The water contact angle of the WPU, WPUA, and UV-cured WPUA film samples rose from 67° to 83°, as illustrated in Figure 7 (C). A decrease in surface free energy was observed as the film transitioned from WPU to WPUA and UV-cured WPUA film. The surface free energy dropped from 35.87 mN/m for WPU, to 29 mN/m after incorporating hydrophobic UDMA, and further to 23.31 mN/m following the UV-curing process. The integration of the hydrophobic UDMA component, along with alterations in contact angle and surface free energy, indicates enhanced water resistance in the coating. This improvement is due to the heightened hydrophobicity of the aqueous polyurethane mixture. After UV curing, the WPUA film exhibits increased hydrophobicity, augmenting its water absorption resistance. This enhancement is presumably attributable to the decreased mobility of polymer chains resulting from crosslinking, which has been corroborated by previous research^54–56^. The findings indicate that UV-cured WPUA coatings enhance mechanical qualities and offer exceptional moisture protection, rendering them optimal for coating applications and prolonging the lifespan of surface coatings.

The cross-cut adhesion (tape adhesion) and pull-off tensile strength tests were conducted to assess the adhesive characteristics of the WPU, WPUA, and UV-cured WPUA films on stainless steel substrates. The obtained findings are displayed in Fig. 7 (D). The cross-cut adhesion test categorizes adhesion performance on a numerical scale ranging from 0B (indicating poor adhesion) to 5B (indicating great adhesion). For the WPU film, the test indicated good adhesion, with 5–15% of the coated area removed, corresponding to a 3B rating. This reflects moderate adhesion between the WPU film and the stainless-steel substrate. The WPUA film was subjected to the same test, both before and after UV curing. Before curing, the WPUA film demonstrated superior adhesion, achieving a 4B rating, with minimal removal of the coating. After curing, the adhesion rating improved to 5B, reflecting excellent adhesion with no coating squares removed^57^. The findings of the cross-cut adhesion test indicate that the inclusion of acrylate groups in UDMA greatly enhances the adherence of polyurethane films to stainless steel, especially after undergoing UV curing. The improvement can be ascribed to the development of a more robust binding between the film and the substrate during the curing procedure. Furthermore, the films underwent a pull-off adhesion test, which demonstrated that the adhesion strength of WPU and WPUA films to stainless steel substrates varied between 8 and 22 kgf/cm². The findings suggest that the inclusion of UDMA prepolymer in the structure of polyurethane acrylate greatly improves the ability to adhere to stainless steel^58^. Furthermore, the process of UV curing of the WPUA film confirms the beneficial impact of UV curing on enhancing the adhesive characteristics and durability of the coating.

## Conclusion

This research effectively synthesized innovative UV-curable waterborne polyurethane acrylates (WPUA) derived from UDMA, utilizing the semi-interpenetrating polymer network (sIPN) approach. A solvent-free WPUA formulation with exceptional water resistance, durability, and adhesion properties has been developed. The WPUA formulations demonstrated good storage stability, with a shelf life exceeding two weeks for WPUA and extended stability for WPU. Particle size exhibited monodisperse distributions and low polydispersity indexes. During the initial week, the WPU demonstrated a particle size of 82 nm and a PDI of 0.1, whereas the WPUA displayed a particle size of 120 nm and a PDI of 0.2. FT-IR analysis confirmed the successful integration of WPU and UDMA components. Additionally, the hydrophobicity of the films increased progressively from WPU to WPUA and UV-cured WPUA, as indicated by an increase in water contact angle from 67° to 83°. The WPUA film showed excellent mechanical properties and water resistance, highlighting the importance of the chemical bonding between WPU and UDMA in enhancing film performance. SEM images taken before and after the curing process revealed a rougher surface, though no phase separation was observed. Adhesion properties were evaluated using cross-cut adhesion (tape adhesion) and pull-off tensile strength tests, showing that the incorporation of UDMA and cross-linking of the double bonds significantly improved the coating’s properties.

## Electronic supplementary material

Below is the link to the electronic supplementary material.

Supplementary Material 1

## Data Availability

Data is provided within the manuscript and supplementary information files.
