# Supplementary material for: Solvent free UV curable waterborne polyurethane acrylate coatings with enhanced hydrophobicity induced by a semi interpenetrating polymer network
Source: Sci Rep. 2025 Jul 1;15:21844. doi: 10.1038/s41598-025-04739-1 (PMC12217702; doi:10.1038/s41598-025-04739-1)
Supplement: Supplementary file 1 — Supplementary Material 1 [file 41598_2025_4739_MOESM1_ESM.docx]

Supporting Information

Solvent Free UV Curable Waterborne Polyurethane Acrylate Coatings with Enhanced Hydrophobicity Induced by a Semi Interpenetrating Polymer Network

Ali Reza Banan*

Department of Organic Chemistry, Imam Hossein University, Tehran, Iran

*Corresponding author: E-mail: bananjahromi@yahoo.com


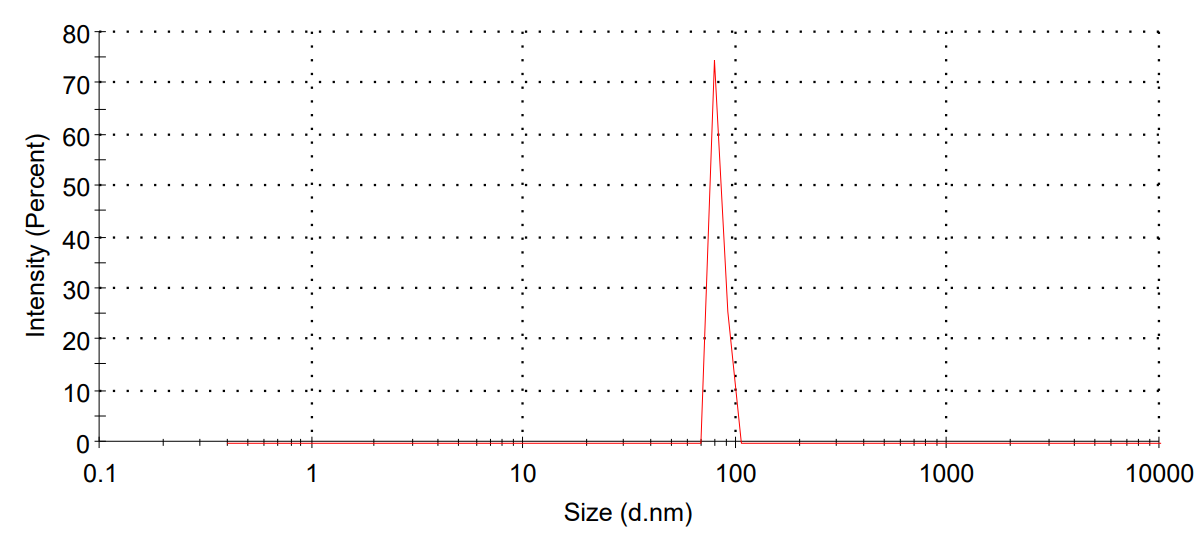


**Figure S1.** WPU particle size in the first week (82nm).


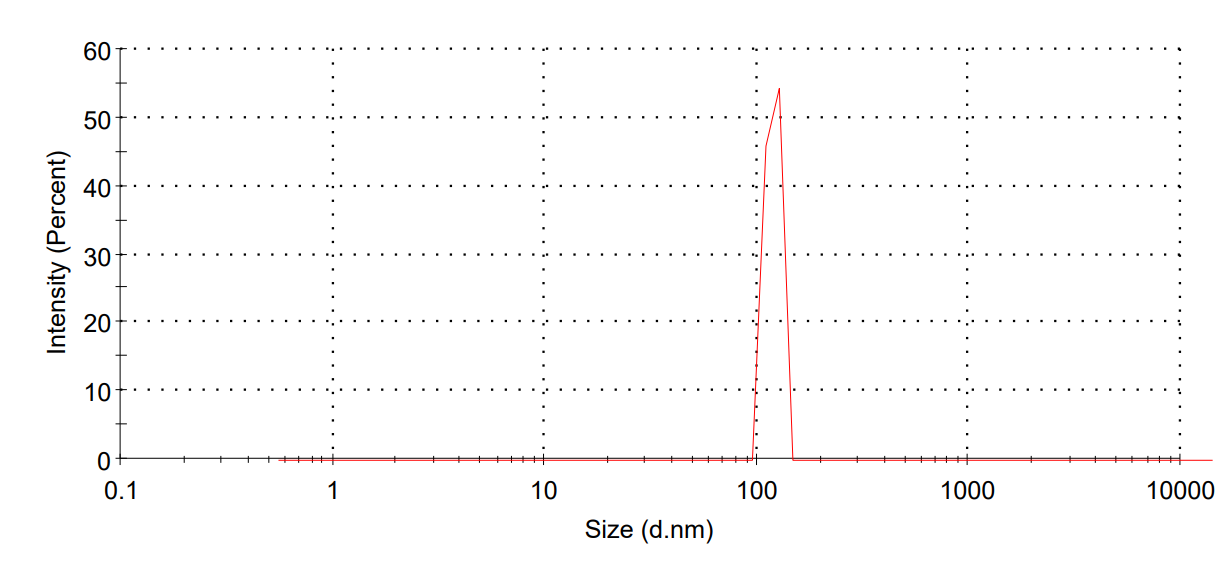


**Figure S2.** WPUA particle size in the first week (120 nm).


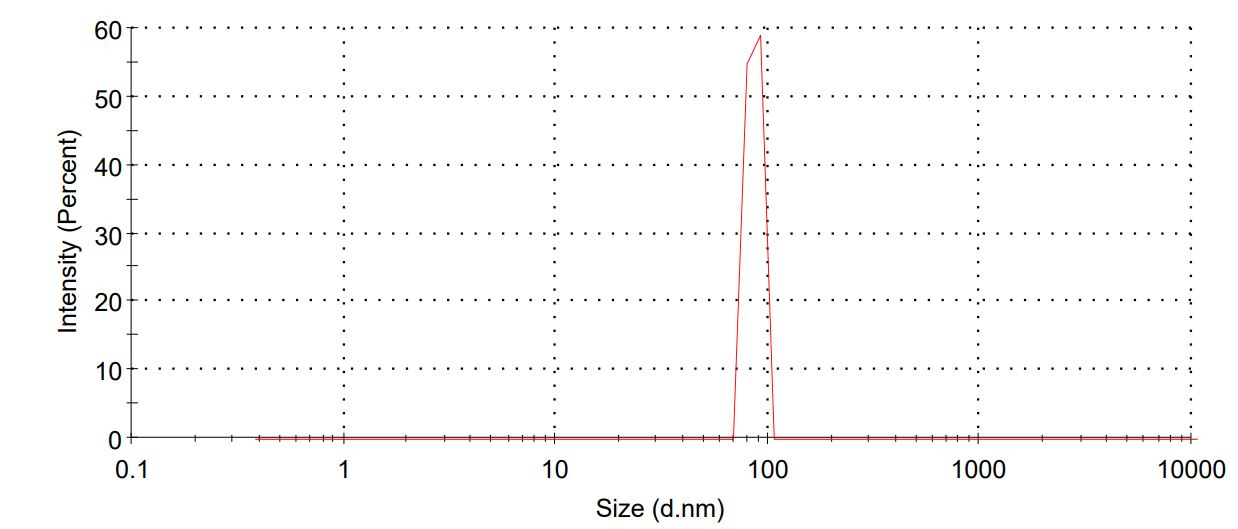


**Figure S3.** WPU particle size in the second week (93nm).


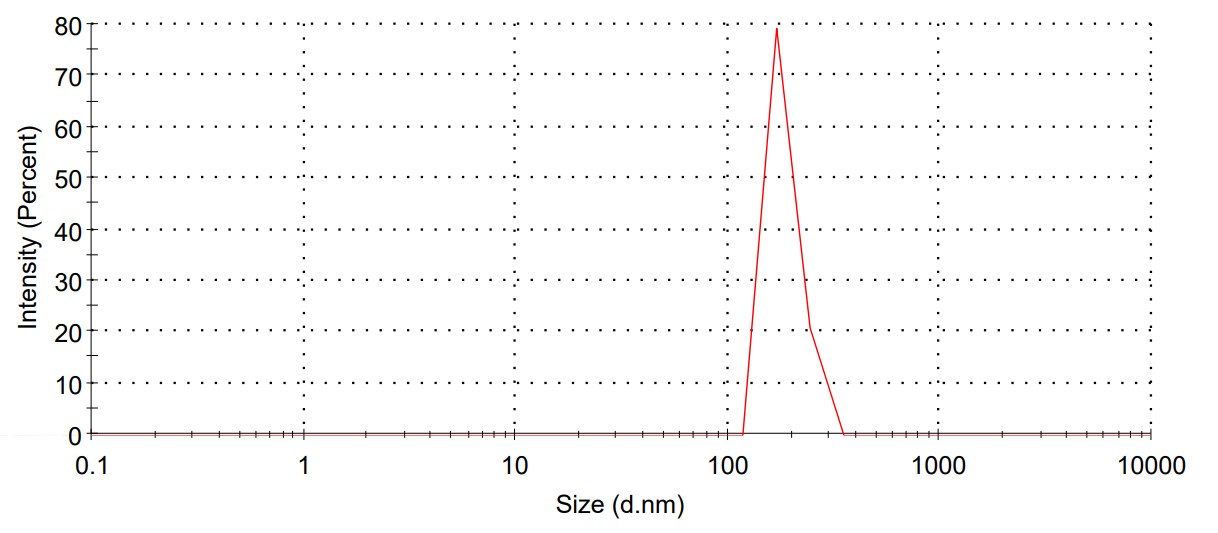


**Figure S4.** WPUA particle size in the second week (132 nm).


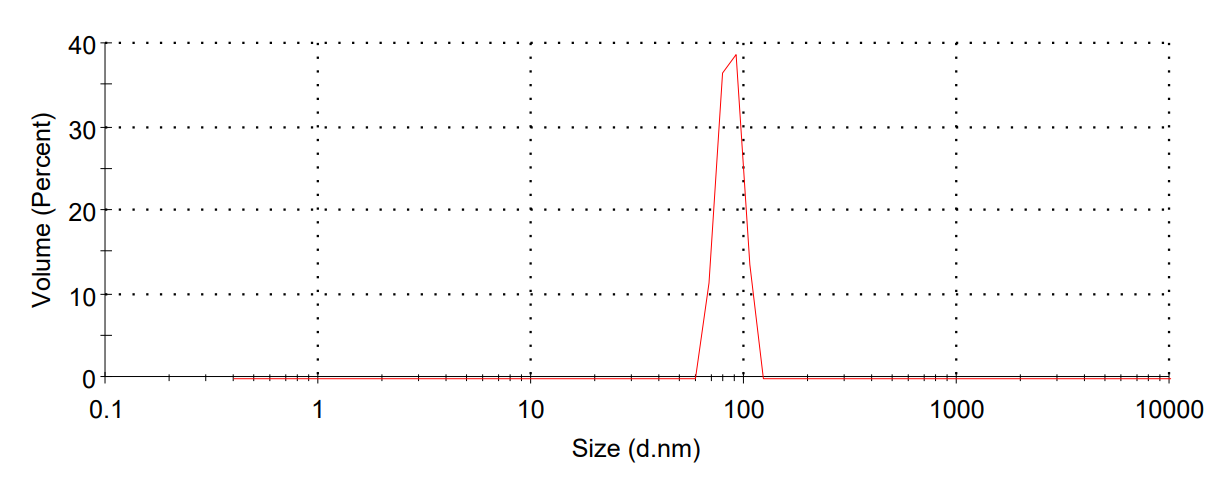


**Figure S4.** WPUA particle size in the third week (98 nm).
